# Supplementary material for: Whole-body microbiota of newborn calves and their response to prenatal vitamin and mineral supplementation
Source: Front Microbiol. 2023 Jun 26;14:1207601. doi: 10.3389/fmicb.2023.1207601 (PMC10331429; doi:10.3389/fmicb.2023.1207601)
Supplement: Supplementary file 1 [file Data_Sheet_1.PDF]

**Supplementary Table S1.** Mineral and vitamin supplement composition.

| **Nutrient** | **Assurance levels** | |
|---|---|---|
| ***Minerals***[1] | **Min** | **Max** |
| Calcium, g / kg of DM | 135.0 | 162.0 |
| Phosphorus, g / kg of DM | 75.0 | - |
| Sodium cholride, g / kg of DM | 180.0 | 216.0 |
| Magnesium, g / kg of DM | 10.0 | - |
| Potassium, g /kg of DM | 10.0 | - |
| Manganese, mg / kg of DM | 3,600.0 | - |
| Cobalt, mg / kg of DM | 12.0 | - |
| Copper, mg / kg of DM | 1200.0 | - |
| Iodine, mg / kg of DM | 60.0 | - |
| Selenium, mg / kg of DM | 27.0 | - |
| Zinc, mg / kg of DM | 3,600.0 | - |
| ***Vitamins, IU / kg of DM*** | | |
| A | 661,500.0 | |
| D | 66,150.0 | |
| E | 661.5 | |

[1]Purina Wind and Rain Storm All Season 7.5 Complete Mineral (Land O' Lakes, Inc., Arden Hills, MN); ingredients: dicalcium phosphate, monocalcium phosphate, processed grain by-products, plant protein products, calcium carbonate, molasses products, salt, mineral oil, potassium chloride, magnesium oxide, ferric oxide, vitamin E supplement, vitamin A supplement, lignin sulfonate, cobalt carbonate, manganese sulfate, ethylenediamine dihydroiodide, zinc sulfate, copper chloride, vitamin D3 supplement, natural and artificial flavors, and sodium selenite. DM = dry matter.

**Supplementary Table S2.** Relative abundance of the top 40 most relatively abundant bacterial genera in ocular, hoof, liver, lung, ruminal tissue, ruminal fluid, vaginal, and nasal microbiota of calves born from dams that received either vitamin and mineral (VTM) supplementation or no VTM supplementation (CON), sampled at 30-h after initial colostrum feeding (n = 7/group).

| **Ocular Swab** | | | | | **Hoof Swab** | | | | |
|---|---|---|---|---|---|---|---|---|---|
| **Genus** | **Rank** | **CON** | **VTM** | **SEM** | **Genus** | **Rank** | **CON** | **VTM** | **SEM** |
| *Streptococcus* | 1 | 67.18 | 48.09 | 9.66 | *Corynebacterium* | 1 | 19.16 | 34.96 | 6.23 |
| *Clostridium sensu stricto 7* | 2 | 2.77 | 6.15 | 1.51 | *Macrococcus* | 2 | 24.23 | 11.75 | 5.22 |
| *Escherichia-Shigella* | 3 | 6.97 | 1.67 | 2.25 | *Facklamia* | 3 | 14.29 | 17.96 | 2.76 |
| *Corynebacterium* | 4 | 2.57 | 3.87 | 1.63 | *Peptostreptococcus* | 4 | 5.93 | 5.96 | 1.85 |
| *Acinetobacter* | 5 | 1.23 | 5.08 | 1.62 | *Staphylococcus* | 5 | 4.71 | 2.98 | 1.84 |
| *Klebsiella* | 6 | 2.18 | 3.95 | 2.11 | *Peptoniphilus* | 6 | 3.98 | 1.42 | 1.20 |
| *Clostridium sensu stricto 1* | 7 | 2.78 | 2.20 | 1.39 | *Gallicola* | 7 | 2.24 | 2.67 | 1.19 |
| *Moraxella* | 8 | 0.73 | 3.07 | 0.62 | *Porphyromonas* | 8 | 0.61 | 2.56 | 1.14 |
| *Jeotgalicoccus* | 9 | 1.54 | 1.94 | 0.85 | *Helcococcus* | 9 | 1.40 | 1.69 | 0.29 |
| *Planococcus* | 10 | 0.96 | 2.21 | 0.85 | *Acinetobacter* | 10 | 1.81 | 1.26 | 0.89 |
| *Bibersteinia* | 11 | 0.99 | 1.33 | 0.28 | *Escherichia-Shigella* | 11 | 0.90 | 1.59 | 0.48 |
| *Solibacillus* | 12 | 0.39 | 1.25 | 0.44 | *Streptococcus* | 12 | 1.36 | 0.86 | 0.36 |
| *Butyricicoccus* | 13 | 0.24 | 1.17 | 0.61 | *Mannheimia* | 13 | 1.84 | 0.16 | 0.98 |
| *Dietzia* | 14 | 0.23 | 0.69 | 0.17 | *W5053* | 14 | 1.09 | 0.63 | 0.46 |
| *Pseudomonas* | 15 | 0.17 | 0.66 | 0.17 | *Ignavigranum* | 15 | 0.72 | 0.94 | 0.20 |
| *Enteractinococcus* | 16 | 0.34 | 0.44 | 0.22 | *Jeotgalicoccus* | 16 | 1.04 | 0.60 | 0.44 |
| *Caryophanon* | 17 | 0.15 | 0.58 | 0.18 | *Salinicoccus* | 17 | 0.81 | 0.36 | 0.17 |
| *Lactobacillus* | 18 | 0.09 | 0.62 | 0.19 | *Clostridium sensu stricto 1* | 18 | 0.39 | 0.68 | 0.23 |
| *Staphylococcus* | 19 | 0.25 | 0.41 | 0.15 | *Globicatella* | 19 | 0.45 | 0.40 | 0.11 |
| *Mannheimia* | 20 | 0.05 | 0.60 | 0.17 | *Rothia* | 20 | 0.69 | 0.09 | 0.34 |
| *Bacillus* | 21 | 0.25 | 0.40 | 0.13 | *Planococcus* | 21 | 0.38 | 0.32 | 0.16 |
| *Conchiformibius* | 22 | 0.13 | 0.50 | 0.08 | *Micrococcus* | 22 | 0.46 | 0.21 | 0.15 |
| *Atopostipes* | 23 | 0.26 | 0.32 | 0.15 | *Klebsiella* | 23 | 0.43 | 0.22 | 0.19 |
| *Ornithinimicrobium* | 24 | 0.13 | 0.41 | 0.12 | *Anaerococcus* | 24 | 0.35 | 0.29 | 0.14 |
| *Lactococcus* | 25 | 0.37 | 0.16 | 0.08 | *Psychrobacter* | 25 | 0.58 | 0.02 | 0.29 |
| *Rothia* | 26 | 0.09 | 0.32 | 0.11 | *Moraxella* | 26 | 0.42 | 0.17 | 0.26 |
| *Ornithinicoccus* | 27 | 0.12 | 0.29 | 0.09 | *Dietzia* | 27 | 0.21 | 0.31 | 0.07 |
| *Comamonas* | 28 | 0.05 | 0.35 | 0.09 | *Enterococcus* | 28 | 0.22 | 0.30 | 0.09 |
| *Glutamicibacter* | 29 | 0.09 | 0.30 | 0.10 | *Solibacillus* | 29 | 0.35 | 0.13 | 0.18 |
| *Luteimonas* | 30 | 0.18 | 0.19 | 0.09 | *Comamonas* | 30 | 0.26 | 0.14 | 0.13 |
| *Brachybacterium* | 31 | 0.16 | 0.17 | 0.07 | *Enteractinococcus* | 31 | 0.12 | 0.24 | 0.07 |

| | | | | | | | | | |
|---|---|---|---|---|---|---|---|---|---|
| *Enterococcus* | 32 | 0.22 | 0.07 | 0.07 | *Proteus* | 32 | 0.15 | 0.21 | 0.07 |
| *Porphyromonas* | 33 | 0.08 | 0.16 | 0.04 | *Conchiformibius* | 33 | 0.24 | 0.08 | 0.13 |
| *Devosia* | 34 | 0.08 | 0.15 | 0.07 | *Clostridium sensu stricto 13* | 34 | 0.09 | 0.16 | 0.07 |
| *Brevundimonas* | 35 | 0.06 | 0.16 | 0.05 | *Caryophanon* | 35 | 0.15 | 0.10 | 0.07 |
| *Jeotgalibaca* | 36 | 0.08 | 0.15 | 0.06 | *Actinobacillus* | 36 | 0.15 | 0.10 | 0.11 |
| *Carnobacterium* | 37 | 0.11 | 0.11 | 0.02 | *Fusobacterium* | 37 | 0.20 | 0.01 | 0.08 |
| *Brevibacterium* | 38 | 0.08 | 0.14 | 0.05 | *Ornithinimicrobium* | 38 | 0.08 | 0.13 | 0.04 |
| *Actinobacillus* | 39 | 0.09 | 0.12 | 0.04 | *Clostridium sensu stricto 7* | 39 | 0.08 | 0.10 | 0.02 |
| *Pedobacter* | 40 | 0.12 | 0.08 | 0.05 | *Pseudomonas* | 40 | 0.04 | 0.14 | 0.04 |

| **Liver Tissue** | | | | | **Lung Tissue** | | | | |
|---|---|---|---|---|---|---|---|---|---|
| **Genus** | **Rank** | **CON** | **VTM** | **SEM** | **Genus** | **Rank** | **CON** | **VTM** | **SEM** |
| *Staphylococcus* | 1 | 39.68 | 43.29 | 7.16 | *Streptococcus* | 1 | 29.75 | 49.20 | 13.00 |
| *Corynebacterium* | 2 | 5.45 | 6.65 | 2.21 | *Staphylococcus* | 2 | 15.61 | 6.53 | 4.52 |
| *Streptococcus* | 3 | 4.94 | 4.37 | 2.44 | *Escherichia-Shigella* | 3 | 5.38 | 4.01 | 1.12 |
| *Bacillus* | 4 | 3.81 | 4.56 | 1.87 | *Corynebacterium* | 4 | 4.83 | 3.36 | 1.76 |
| *Acinetobacter* | 5 | 1.93 | 5.65 | 1.89 | *Acinetobacter* | 5 | 3.96 | 2.91 | 0.93 |
| *Escherichia-Shigella* | 6 | 4.10 | 0.32 | 1.38 | *Klebsiella* | 6 | 2.34 | 4.42 | 1.55 |
| *Klebsiella* | 7 | 3.01 | 0.33 | 1.09 | *Bacillus* | 7 | 2.71 | 3.01 | 2.60 |
| *Clostridium sensu stricto 1* | 8 | 3.27 | 0.00 | 1.11 | *Clostridium sensu stricto 1* | 8 | 2.86 | 2.56 | 1.67 |
| *Micrococcus* | 9 | 1.03 | 1.79 | 1.03 | *Butyricicoccus* | 9 | 1.77 | 0.82 | 1.05 |
| *Enhydrobacter* | 10 | 2.58 | 0.03 | 0.65 | *Geobacillus* | 10 | 0.10 | 2.37 | 1.24 |
| *Flavobacterium* | 11 | 1.09 | 1.23 | 0.46 | *Planococcus* | 11 | 2.21 | 0.18 | 0.63 |
| *Ileibacterium* | 12 | 0.00 | 1.95 | 0.90 | *Bibersteinia* | 12 | 0.81 | 0.88 | 0.43 |
| *Blautia* | 13 | 0.72 | 1.19 | 0.96 | *Moraxella* | 13 | 0.60 | 1.09 | 0.44 |
| *Arsenicicoccus* | 14 | 1.05 | 0.85 | 0.95 | *Enterococcus* | 14 | 0.06 | 1.59 | 0.81 |
| *Faecalibacterium* | 15 | 1.84 | 0.00 | 0.92 | *Pseudomonas* | 15 | 1.02 | 0.53 | 0.47 |
| *Pandoraea* | 16 | 0.78 | 0.84 | 0.57 | *Jeotgalicoccus* | 16 | 1.14 | 0.16 | 0.33 |
| *Ralstonia* | 17 | 0.66 | 0.92 | 0.48 | *Solibacillus* | 17 | 1.14 | 0.15 | 0.36 |
| *Haemophilus* | 18 | 0.23 | 1.15 | 0.46 | *Lactobacillus* | 18 | 0.39 | 0.73 | 0.41 |
| *Anaerococcus* | 19 | 0.41 | 0.83 | 0.41 | *[Ruminococcus] gnavus group* | 19 | 0.46 | 0.59 | 0.47 |
| *Solibacillus* | 20 | 0.00 | 1.09 | 0.54 | *Pandoraea* | 20 | 0.58 | 0.40 | 0.33 |
| *Anoxybacillus* | 21 | 0.00 | 1.08 | 0.51 | *Finegoldia* | 21 | 0.83 | 0.01 | 0.26 |
| *Lautropia* | 22 | 0.00 | 1.08 | 0.54 | *Blautia* | 22 | 0.00 | 0.72 | 0.21 |
| *Sediminibacterium* | 23 | 0.23 | 0.83 | 0.51 | *Rheinheimera* | 23 | 0.65 | 0.05 | 0.30 |
| *Hydrogenophaga* | 24 | 0.96 | 0.14 | 0.55 | *Haemophilus* | 24 | 0.62 | 0.06 | 0.32 |
| *Arthrobacter* | 25 | 0.00 | 0.85 | 0.40 | *Thermicanus* | 25 | 0.00 | 0.66 | 0.26 |
| *ADurb.Bin063-1* | 26 | 1.00 | 0.00 | 0.44 | *Micrococcus* | 26 | 0.41 | 0.25 | 0.31 |
| *Candidatus Udaeobacter* | 27 | 0.99 | 0.00 | 0.50 | *Anaerococcus* | 27 | 0.29 | 0.36 | 0.25 |
| *Neisseria* | 28 | 0.93 | 0.05 | 0.49 | *Mannheimia* | 28 | 0.01 | 0.63 | 0.29 |

| | | | | | | | | | |
|---|---|---|---|---|---|---|---|---|---|
| *Luminiphilus* | 29 | 0.00 | 0.82 | 0.41 | *Comamonas* | 29 | 0.35 | 0.24 | 0.19 |
| *Enterococcus* | 30 | 0.63 | 0.27 | 0.26 | *Succinivibrionaceae UCG-001* | 30 | 0.57 | 0.00 | 0.29 |
| *Bacteroides* | 31 | 0.00 | 0.79 | 0.39 | *Peptoclostridium* | 31 | 0.57 | 0.00 | 0.16 |
| *Planococcus* | 32 | 0.28 | 0.52 | 0.37 | *Lactococcus* | 32 | 0.33 | 0.22 | 0.13 |
| *Serratia* | 33 | 0.88 | 0.00 | 0.41 | *Actinobacillus* | 33 | 0.34 | 0.22 | 0.19 |
| *Candidatus Limnoluna* | 34 | 0.78 | 0.08 | 0.37 | *Paracoccus* | 34 | 0.31 | 0.19 | 0.18 |
| *Megasphaera* | 35 | 0.86 | 0.00 | 0.43 | *Moheibacter* | 35 | 0.27 | 0.23 | 0.25 |
| *Paucibacter* | 36 | 0.77 | 0.03 | 0.39 | *Gallibacterium* | 36 | 0.46 | 0.00 | 0.23 |
| *Nocardioides* | 37 | 0.00 | 0.69 | 0.34 | *Blastococcus* | 37 | 0.44 | 0.00 | 0.22 |
| *Ochrobactrum* | 38 | 0.81 | 0.00 | 0.40 | *Bifidobacterium* | 38 | 0.00 | 0.44 | 0.17 |
| *Thermicanus* | 39 | 0.00 | 0.68 | 0.24 | *Anoxybacillus* | 39 | 0.00 | 0.41 | 0.20 |
| *Pelomonas* | 40 | 0.32 | 0.39 | 0.30 | *Fusobacterium* | 40 | 0.40 | 0.01 | 0.20 |

| **Ruminal Tissue** | | | | | **Ruminal Fluid** | | | | |
|---|---|---|---|---|---|---|---|---|---|
| **Genus** | **Rank** | **CON** | **VTM** | **SEM** | **Genus** | **Rank** | **CON** | **VTM** | **SEM** |
| *Streptococcus* | 1 | 31.51 | 35.26 | 5.47 | *Streptococcus* | 1 | 75.57 | 85.37 | 2.30 |
| *Klebsiella* | 2 | 14.72 | 14.35 | 3.63 | *Escherichia-Shigella* | 2 | 14.05 | 3.60 | 1.95 |
| *Escherichia-Shigella* | 3 | 18.73 | 8.33 | 1.98 | *Klebsiella* | 3 | 4.61 | 5.88 | 1.71 |
| *Clostridium sensu stricto 1* | 4 | 12.67 | 3.29 | 4.39 | *Lactobacillus* | 4 | 0.11 | 2.34 | 1.11 |
| *Acinetobacter* | 5 | 4.48 | 7.75 | 2.27 | *Acinetobacter* | 5 | 1.46 | 0.55 | 0.23 |
| *Butyricicoccus* | 6 | 3.97 | 2.93 | 2.46 | *Gallibacterium* | 6 | 1.63 | 0.01 | 0.81 |
| *Staphylococcus* | 7 | 0.59 | 4.60 | 1.03 | *Bibersteinia* | 7 | 0.69 | 0.73 | 0.11 |
| *Bacillus* | 8 | 1.21 | 1.61 | 1.28 | *Lactococcus* | 8 | 0.43 | 0.39 | 0.06 |
| *Gallibacterium* | 9 | 2.47 | 0.00 | 1.23 | *Moraxella* | 9 | 0.34 | 0.28 | 0.09 |
| *Bibersteinia* | 10 | 1.27 | 1.04 | 0.33 | *Carnobacterium* | 10 | 0.18 | 0.18 | 0.04 |
| *Moraxella* | 11 | 0.95 | 1.17 | 0.52 | *Leuconostoc* | 11 | 0.15 | 0.11 | 0.03 |
| *Lactococcus* | 12 | 0.89 | 1.15 | 0.21 | *Mannheimia* | 12 | 0.03 | 0.13 | 0.06 |
| *Lactobacillus* | 13 | 0.64 | 1.08 | 0.32 | *Enterococcus* | 13 | 0.13 | 0.01 | 0.05 |
| *Corynebacterium* | 14 | 0.28 | 1.25 | 0.23 | *Porphyromonas* | 14 | 0.082 | 0.051 | 0.03 |
| *Leuconostoc* | 15 | 0.49 | 0.70 | 0.16 | *HT002* | 15 | 0.045 | 0.079 | 0.04 |
| *Carnobacterium* | 16 | 0.39 | 0.29 | 0.10 | *Actinobacillus* | 16 | 0.060 | 0.055 | 0.01 |
| *Sarcina* | 17 | 0.14 | 0.44 | 0.28 | *Conchiformibius* | 17 | 0.045 | 0.043 | 0.01 |
| *Peptoclostridium* | 18 | 0.03 | 0.54 | 0.27 | *Pelistega* | 18 | 0.081 | 0.000 | 0.04 |
| *Conchiformibius* | 19 | 0.18 | 0.35 | 0.12 | *Ligilactobacillus* | 19 | 0.059 | 0.000 | 0.02 |
| *Clostridium sensu stricto 2* | 20 | 0.34 | 0.18 | 0.20 | *Bergeyella* | 20 | 0.026 | 0.033 | 0.01 |
| *[Ruminococcus] gnavus group* | 21 | 0.22 | 0.29 | 0.19 | *Weissella* | 21 | 0.049 | 0.000 | 0.01 |
| *Planococcus* | 22 | 0.06 | 0.44 | 0.16 | *Fructobacillus* | 22 | 0.020 | 0.021 | 0.01 |
| *Pseudomonas* | 23 | 0.05 | 0.45 | 0.15 | *Kosakonia* | 23 | 0.029 | 0.000 | 0.01 |
| *Alkanindiges* | 24 | 0.00 | 0.37 | 0.17 | *Alkanindiges* | 24 | 0.007 | 0.021 | 0.01 |
| *Mannheimia* | 25 | 0.07 | 0.28 | 0.13 | *Rothia* | 25 | 0.007 | 0.015 | 0.00 |
| *Actinobacillus* | 26 | 0.14 | 0.22 | 0.08 | *Comamonas* | 26 | 0.018 | 0.001 | 0.01 |

| | | | | | | | | | |
|---|---|---|---|---|---|---|---|---|---|
| *Ligilactobacillus* | 27 | 0.18 | 0.17 | 0.15 | *Limosilactobacillus* | 27 | 0.003 | 0.012 | 0.01 |
| *Tepidiphilus* | 28 | 0.06 | 0.28 | 0.17 | *Alysiella* | 28 | 0.006 | 0.007 | 0.004 |
| *Flavobacterium* | 29 | 0.01 | 0.33 | 0.16 | *Pseudomonas* | 29 | 0.006 | 0.006 | 0.003 |
| *Anaerococcus* | 30 | 0.01 | 0.31 | 0.09 | *Bacillus* | 30 | 0.009 | 0.003 | 0.003 |
| *Micrococcus* | 31 | 0.02 | 0.29 | 0.06 | *Bifidobacterium* | 31 | 0.009 | 0.001 | 0.003 |
| *Epulopiscium* | 32 | 0.26 | 0.04 | 0.14 | *Stenotrophomonas* | 32 | 0.007 | 0.001 | 0.003 |
| *Fructobacillus* | 33 | 0.12 | 0.16 | 0.07 | *Clostridium sensu stricto 1* | 33 | 0.007 | 0.001 | 0.003 |
| *Pelistega* | 34 | 0.28 | 0.00 | 0.14 | *Corynebacterium* | 34 | 0.003 | 0.002 | 0.002 |
| *Porphyromonas* | 35 | 0.22 | 0.06 | 0.07 | *Liquorilactobacillus* | 35 | 0.003 | 0.002 | 0.002 |
| *Comamonas* | 36 | 0.10 | 0.18 | 0.08 | *Kurthia* | 36 | 0.002 | 0.002 | 0.001 |
| *Bifidobacterium* | 37 | 0.12 | 0.16 | 0.13 | *Staphylococcus* | 37 | 0.000 | 0.004 | 0.001 |
| *Rothia* | 38 | 0.03 | 0.24 | 0.08 | *Thermus* | 38 | 0.001 | 0.002 | 0.001 |
| *Candidatus Udaeobacter* | 39 | 0.00 | 0.24 | 0.12 | *Planococcus* | 39 | 0.001 | 0.002 | 0.001 |
| *Dietzia* | 40 | 0.01 | 0.24 | 0.10 | *Gemella* | 40 | 0.002 | 0.000 | 0.001 |

| **Vaginal Swab** | | | | | **Nasal Swab** | | | | |
|---|---|---|---|---|---|---|---|---|---|
| **Genus** | **Rank** | **CON** | **VTM** | **SEM** | **Genus** | **Rank** | **CON** | **VTM** | **SEM** |
| *Streptococcus* | 1 | 39.20 | 26.99 | 7.32 | *Streptococcus* | 1 | 65.59 | 59.22 | 15.14 |
| *Clostridium sensu stricto 1* | 2 | 14.71 | 7.78 | 3.08 | *Acinetobacter* | 2 | 0.89 | 5.80 | 2.65 |
| *Escherichia-Shigella* | 3 | 13.61 | 8.75 | 4.13 | *Conchiformibius* | 3 | 5.83 | 0.30 | 2.86 |
| *Staphylococcus* | 4 | 0.40 | 19.39 | 5.76 | *Moraxella* | 4 | 1.45 | 4.35 | 1.91 |
| *Klebsiella* | 5 | 9.79 | 5.38 | 3.03 | *Bibersteinia* | 5 | 1.76 | 2.42 | 1.00 |
| *Bibersteinia* | 6 | 9.51 | 0.07 | 4.76 | *Staphylococcus* | 6 | 3.19 | 0.56 | 1.60 |
| *Butyricicoccus* | 7 | 1.63 | 4.75 | 1.99 | *Escherichia-Shigella* | 7 | 2.50 | 1.17 | 0.52 |
| *Acinetobacter* | 8 | 0.58 | 4.56 | 1.45 | *Klebsiella* | 8 | 1.79 | 1.47 | 0.65 |
| *Corynebacterium* | 9 | 0.62 | 2.96 | 0.54 | *Corynebacterium* | 9 | 0.90 | 2.25 | 1.09 |
| *Actinobacillus* | 10 | 2.51 | 0.94 | 1.64 | *Mannheimia* | 10 | 1.82 | 1.16 | 1.22 |
| *Pseudomonas* | 11 | 0.07 | 2.30 | 0.98 | *Planococcus* | 11 | 1.74 | 1.00 | 1.00 |
| *Mannheimia* | 12 | 1.10 | 0.96 | 0.80 | *Rothia* | 12 | 0.37 | 2.00 | 0.89 |
| *Planococcus* | 13 | 0.56 | 1.24 | 0.40 | *Jeotgalicoccus* | 13 | 0.55 | 0.83 | 0.40 |
| *Rothia* | 14 | 0.72 | 0.71 | 0.41 | *Clostridium sensu stricto 7* | 14 | 0.26 | 0.73 | 0.35 |
| *Jeotgalicoccus* | 15 | 0.27 | 1.06 | 0.18 | *Lactobacillus* | 15 | 0.08 | 0.69 | 0.29 |
| *Enterococcus* | 16 | 0.55 | 0.31 | 0.18 | *Clostridium sensu stricto 1* | 16 | 0.41 | 0.34 | 0.26 |
| *Epulopiscium* | 17 | 0.63 | 0.18 | 0.36 | *Porphyromonas* | 17 | 0.42 | 0.17 | 0.22 |
| *Clostridium sensu stricto 7* | 18 | 0.11 | 0.63 | 0.23 | *Actinobacillus* | 18 | 0.24 | 0.22 | 0.11 |
| *Dietzia* | 19 | 0.12 | 0.56 | 0.15 | *Pseudomonas* | 19 | 0.08 | 0.28 | 0.10 |
| *Terrisporobacter* | 20 | 0.34 | 0.19 | 0.14 | *Bacillus* | 20 | 0.15 | 0.20 | 0.11 |
| *Solibacillus* | 21 | 0.17 | 0.32 | 0.09 | *Lactococcus* | 21 | 0.17 | 0.17 | 0.05 |
| *Ornithinimicrobium* | 22 | 0.14 | 0.34 | 0.12 | *Solibacillus* | 22 | 0.04 | 0.30 | 0.09 |
| *Clostridium sensu stricto 2* | 23 | 0.22 | 0.22 | 0.18 | *Dietzia* | 23 | 0.12 | 0.17 | 0.09 |

| | | | | | | | | | |
|---|---|---|---|---|---|---|---|---|---|
| *Enteractinococcus* | 24 | 0.08 | 0.31 | 0.09 | *Psychrobacter* | 24 | 0.22 | 0.05 | 0.13 |
| *Bacillus* | 25 | 0.10 | 0.28 | 0.10 | *Brevibacterium* | 25 | 0.04 | 0.23 | 0.08 |
| *Ornithinicoccus* | 26 | 0.15 | 0.23 | 0.11 | *Atopostipes* | 26 | 0.11 | 0.15 | 0.08 |
| *Moraxella* | 27 | 0.02 | 0.32 | 0.12 | *Frederiksenia* | 27 | 0.25 | 0.00 | 0.12 |
| *Comamonas* | 28 | 0.11 | 0.19 | 0.08 | *Glutamicibacter* | 28 | 0.12 | 0.12 | 0.12 |
| *[Ruminococcus] gnavus group* | 29 | 0.10 | 0.16 | 0.07 | *Carnobacterium* | 29 | 0.12 | 0.11 | 0.04 |
| *Brachybacterium* | 30 | 0.03 | 0.19 | 0.04 | *Enteractinococcus* | 30 | 0.12 | 0.08 | 0.06 |
| *Caryophanon* | 31 | 0.06 | 0.16 | 0.04 | *Caryophanon* | 31 | 0.02 | 0.16 | 0.06 |
| *Brevibacterium* | 32 | 0.01 | 0.20 | 0.04 | *Bergeyella* | 32 | 0.13 | 0.05 | 0.07 |
| *Glutamicibacter* | 33 | 0.01 | 0.19 | 0.07 | *Enterococcus* | 33 | 0.06 | 0.12 | 0.07 |
| *Leucobacter* | 34 | 0.01 | 0.18 | 0.04 | *Leuconostoc* | 34 | 0.08 | 0.10 | 0.04 |
| *Atopostipes* | 35 | 0.04 | 0.14 | 0.03 | *Comamonas* | 35 | 0.03 | 0.13 | 0.04 |
| *Paracoccus* | 36 | 0.03 | 0.14 | 0.05 | *Brachybacterium* | 36 | 0.06 | 0.07 | 0.05 |
| *Sphingobacterium* | 37 | 0.03 | 0.13 | 0.03 | *Ornithinimicrobium* | 37 | 0.07 | 0.05 | 0.04 |
| *Citricoccus* | 38 | 0.05 | 0.09 | 0.03 | *Pedobacter* | 38 | 0.05 | 0.08 | 0.05 |
| *Alishewanella* | 39 | 0.03 | 0.11 | 0.04 | *Uruburuella* | 39 | 0.04 | 0.07 | 0.04 |
| *Blautia* | 40 | 0.01 | 0.12 | 0.04 | *Georgenia* | 40 | 0.02 | 0.09 | 0.04 |

**Supplementary Table S3**. Average number of sequencing reads (±SEM) before and after removing removal of mitochondria, chloroplast, and negative control sequences.

| **Sample type** | **Sequencing reads before removal of potential contaminant sequencing reads** | **Sequencing reads after removal of potential contaminant sequencing reads** |
|---|---|---|
| Hoof swab | 185904 ± 9970 | 185261 ± 10017 |
| Liver tissue | 232797 ± 18164 | 89852 ± 10847 |
| Lung tissue | 484801 ± 38142 | 369539 ± 42475 |
| Nasal swab | 257610 ± 31260 | 254352 ± 32171 |
| Ocular swab | 272472 ± 20448 | 264921 ± 20423 |
| Ruminal fluid | 276086 ± 16693 | 275955 ± 16704 |
| Ruminal tissue | 432122 ± 26428 | 400929 ± 30425 |
| Vaginal swab | 225180 ± 14767 | 223692 ± 15009 |

**Supplementary Table S4**. PERMANOVA analysis summary for comparison of microbial community structure between different sampling type.

| PERMANOVA | $R^2$ | Adjusted *P - value* | PERMANOVA | $R^2$ | Adjusted *P - value* |
|---|---|---|---|---|---|
| Ruminal fluid vs. Hoof swab | 0.64 | 0.0028 | Liver tissue vs. Vaginal swab | 0.301 | 0.0028 |
| Ruminal fluid vs. Liver tissue | 0.54 | 0.0028 | Liver tissue vs. Ocular swab | 0.434 | 0.0028 |
| Ruminal fluid vs. Ruminal tissue | 0.33 | 0.0028 | Ruminal tissue vs. Vaginal swab | 0.121 | 0.0028 |
| Ruminal fluid vs. Vaginal swab | 0.25 | 0.0028 | Ruminal tissue vs. Ocular swab | 0.257 | 0.0028 |
| Nasal swab vs. Hoof swab | 0.41 | 0.0028 | Ruminal fluid vs. Lung tissue | 0.181 | 0.0448 |
| Nasal swab vs. Liver tissue | 0.35 | 0.0028 | Ruminal fluid vs. Ocular swab | 0.172 | 0.0168 |
| Nasal swab vs. Ruminal tissue | 0.23 | 0.0028 | Nasal swab vs. Vaginal swab | 0.142 | 0.0448 |
| Hoof swab vs. Lung tissue | 0.39 | 0.0028 | Lung tissue vs. Ruminal tissue | 0.140 | 0.0168 |
| Hoof swab vs. Liver tissue | 0.45 | 0.0028 | Vaginal swab vs. Ocular swab | 0.147 | 0.0196 |
| Hoof swab vs. Ruminal tissue | 0.52 | 0.0028 | Ruminal fluid vs. Nasal swab | 0.127 | 0.493 |
| Hoof swab vs. Vaginal swab | 0.38 | 0.0028 | Nasal swab vs. Lung tissue | 0.096 | 0.580 |
| Hoof swab vs. Ocular swab | 0.51 | 0.0028 | Nasal swab vs. Ocular swab | 0.053 | 1.00 |
| Lung tissue vs. Liver tissue | 0.20 | 0.0028 | Lung tissue vs. Vaginal swab | 0.099 | 0.11 |
| Liver tissue vs. Ruminal tissue | 0.40 | 0.0028 | Lung tissue vs. Ocular swab | 0.123 | 0.11 |

**Supplementary Table S5**. Percent relative abundance (%) of these bacterial genera that have been reported to encompasses species associated with bovine infectious diseases*.

| Genus | Rank | CON | VTM | SEM |
|---|---|---|---|---|
| **Ocular swab** | | | | |
| *Moraxella* | 8 | 0.73 | 3.07 | 0.62 |
| *Mannheimia* | 20 | 0.05 | 0.6 | 0.17 |
| *Fusobacterium* | 255 | 0.005 | 0.003 | 0.004 |
| *Trueperella* | 301 | 0 | 0.006 | 0.003 |
| *Histophilus* | 372 | 0 | 0.003 | 0.001 |
| **Liver tissue** | | | | |
| *Pandoraea* | 16 | 0.78 | 0.84 | 0.57 |
| *Haemophilus* | 18 | 0.23 | 1.15 | 0.46 |
| *Megasphaera* | 35 | 0.86 | 0 | 0.43 |
| *Ochrobactrum* | 38 | 0.81 | 0 | 0.4 |
| *Thermicanus* | 39 | 0 | 0.68 | 0.24 |
| *Pelomonas* | 40 | 0.32 | 0.39 | 0.3 |
| **Nasal swab** | | | | |
| *Moraxella* | 4 | 1.45 | 4.35 | 1.91 |
| *Mannheimia* | 10 | 1.82 | 1.16 | 1.22 |
| *Fusobacterium* | 121 | 0.017 | 0 | 0.008 |
| **Vaginal swab** | | | | |
| *Mannheimia* | 12 | 1.1 | 0.96 | 0.8 |
| *Moraxella* | 27 | 0.02 | 0.32 | 0.12 |
| *Fusobacterium* | 361 | 0.0004 | 0.0007 | 0.0004 |
| *Trueperella* | 447 | 0.0004 | 0 | 0.0002 |
| **Hoof swab** | | | | |
| *Mannheimia* | 13 | 1.84 | 0.16 | 0.98 |
| *Moraxella* | 26 | 0.42 | 0.17 | 0.26 |
| *Fusobacterium* | 37 | 0.2 | 0.01 | 0.08 |
| *Trueperalla* | 79 | 0.03 | 0.005 | 0.015 |
| **Lung tissue** | | | | |
| *Moraxella* | 13 | 0.6 | 1.09 | 0.44 |
| *Mannheimia* | 28 | 0.01 | 0.63 | 0.29 |
| *Fusobacterium* | 40 | 0.4 | 0.01 | 0.2 |
| *Trueperella* | 228 | 0.009 | 0 | 0.005 |
| **Rumen fluid** | | | | |
| *Moraxella* | 9 | 0.34 | 0.28 | 0.09 |
| *Mannheimia* | 12 | 0.03 | 0.13 | 0.06 |
| **Rumen tissue** | | | | |
| *Moraxella* | 11 | 0.95 | 1.17 | 0.52 |
| *Mannheimia* | 25 | 0.07 | 0.28 | 0.13 |
| *Fusobacterium* | 163 | 0.015 | 0 | 0.0075 |

*CON: calves born from non-VTM supplemented dams (n = 7); VTM: calves born from VTM supplemented dams (n =7).  SEM: standard error of the mean.

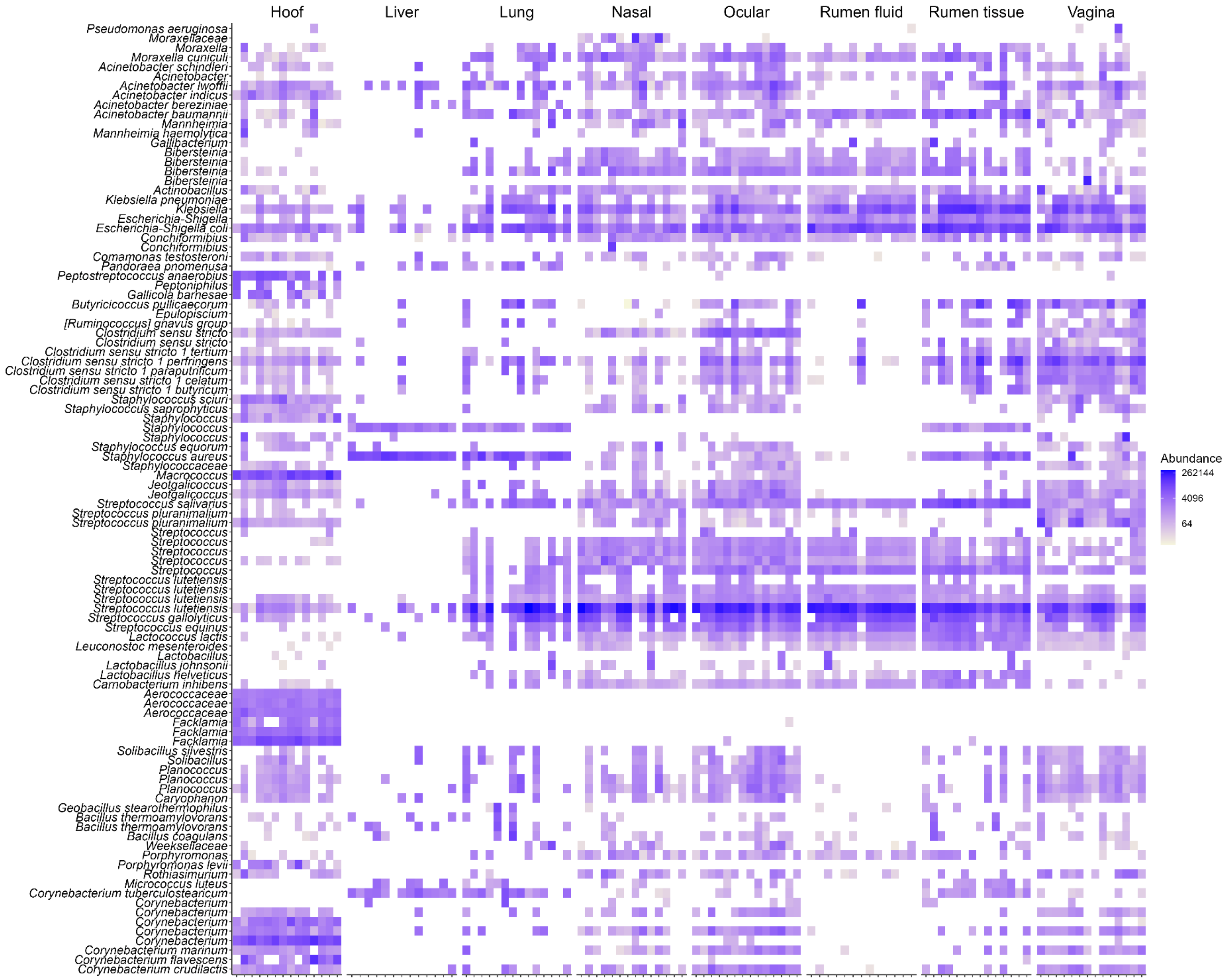


**Supplementary Figure S1**. Heatmap showing the 100 most abundant ASVs (log4) overall sample type.
